# Vascularised fibula osteocutaneous flap for cervical spinal and posterior pharyngeal wall reconstruction

**DOI:** 10.4103/0970-0358.59294

**Published:** 2009

**Authors:** Krishnakumar Thankappan, Sandip Duarah, Nirav P. Trivedi, Dilip Panikar, Moni Abraham Kuriakose, Subramania Iyer

**Affiliations:** Department of Head and Neck Surgery, Amrita Institute of Medical Sciences, Elamakkara, Kochi, India; 1 Department of Neurosurgery, Amrita Institute of Medical Sciences, Elamakkara, Kochi, India

**Keywords:** Free fibula flap, head and neck reconstruction, cervical spinal reconstruction, skull base chordoma, mandibulotomy approach

## Abstract

We report a case of vascularised fibula osteocutaneous flap used for composite cervical spinal and posterior pharyngeal wall reconstruction, in a patient with recurrent skull base chordoma, resected by an anterior approach via median labio-mandibular glossotomy approach. Bone stability and pharyngeal wall integrity were simultaneously restored

## INTRODUCTION

Microvascular transfer of fibular bone has been used in many ways in reconstructive surgery. A case of skull base chordoma, which recurred multiple times, was resected by an anterior approach via median labio-mandibular glossotomy approach. Vascularised fibula osteocutaneous flap was used for cervical spinal and posterior pharyngeal wall reconstruction. The case demonstrates the complexity in the management of recurrent skull base chordomas, and the aptness of the flap in simultaneously restoring the bone stability and pharyngeal wall integrity.

## CASE REPORT

**Figure 1:**
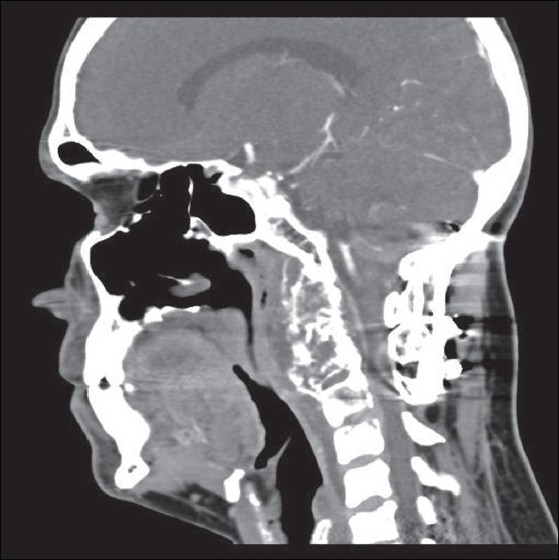
CT Scan (sagital view) showing the lesion

**Figure 2:**
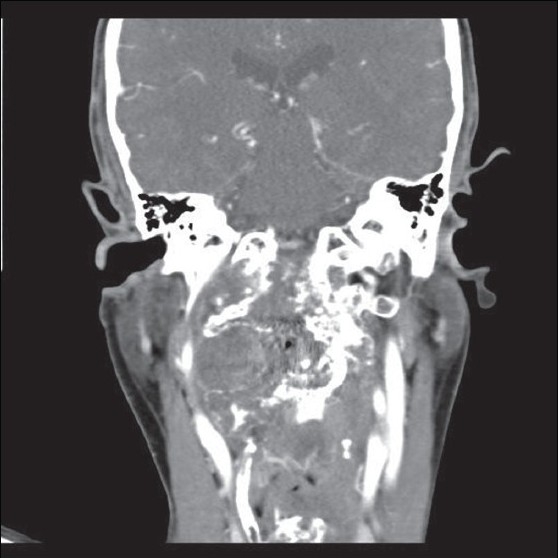
CT Scan (coronal view) showing the lesion

A 41-year-old male with a history of chordoma of the cranio-vertebral junction operated upon multiple times earlier, presented with complaints of pain, limitation of neck movements, and difficulty in swallowing. The first surgery was done eight years back through a posterior approach, at which time the tumour was decompressed and an occipito-cervical stabilization was done. This was followed by transoral decompressions done on two occasions. Neurological examination revealed exaggerated deep tendon reflexes and extensor plantar reflex on the right side with intact cranial nerve functions, normal muscle bulk and power in all four limbs. A computed tomography (CT) scan showed a large recurrence [Figures 1 and 2] involving second and third cervical vertebral bodies, anterior arch and right lateral mass of atlas, right occipital condyle, anterior lip of foramen magnum and lower half of clivus. Right internal carotid artery was displaced anterolaterally and right vertebral artery was encased and pinched off by the tumour. Sub-arachnoid space C1-C3 was totally effaced, and significant secondary narrowing of the spinal canal was present.

**Figure 3:**
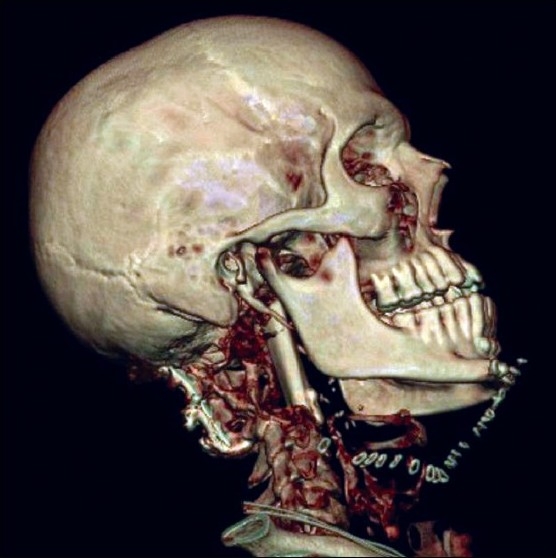
Postoperative three-dimensional CT image

After necessary preoperative evaluation and counselling he underwent a sub total excision of the tumour by median labio-mandibular glossotomy approach. Reconstruction was carried out using a vascularised fibula osteocutaneous flap. The fibular strut was used to reconstruct the vertebral column. It was wedged between healthy bone of the lower clivus and the C3 vertebral body, and held in place with a small mini-plate at the lower end of the graft. The skin paddle was used to reconstruct the posterior pharyngeal wall which was largely scarred from the previous surgeries and was excised with the tumour. The artery was anastomosed to the lingual artery and the vein to one of the tributaries of internal jugular vein in the neck. The recipient vessels in the neck were in close proximity to the donor vessels and so the length of the vascular pedicle was adequate. Figure 3 shows the three-dimensional CT image after surgery. The skin paddle was periodically monitored directly in the oropharynx. Since the manoeuvre of depressing the tongue and visualizing the flap was stressful to the patient due to the presence of the wounds and mandibulotomy, this was done less frequently than the usual hourly observation. Postoperatively, tracheostomy could be removed and satisfactory oral swallowing could be initiated by six weeks. He was treated with adjuvant intensity modulated radiotherapy with 6 MV photons with a dose of 60Gy in 30 fractions, which was tolerated well. After 18 months of follow-up he is symptomatically well, though imaging studies revealed residual disease.

## DISCUSSION

Chordomas are comparatively slow growing malignant neoplasms derived from notochord. In the cranial region the tumours usually arise from the clivus. Clival chordomas usually present in the third and fourth decades of life and there is slight male preponderance. Surgical resection with or without adjuvant radiotherapy is the treatment of choice. Because of their locally invasive nature into the cervical spine, parasellar structures, and intradural extensions, true microscopic total removal of clival chordomas is typically not possible.[1] The endoscopic trans-nasal trans-sphenoidal approach is described for smaller lesions.[2] A maxillo-mandibular swing with a glossotomy has been suggested to be a better approach for larger lesions.[3]

Non vascularised bone grafts have been used successfully for the reconstruction of cervical spine. Iliac crest bones, autogenous and allogeneic segments of fibula have been reported.[4,5] The success of these grafts relies on the healing capacity of the recipient bed. They heal by creeping substitution. This can take up to two years.[6,7] Even with radiographic evidence of incorporation into the adjacent bone, distant portion of the graft may remain avascular and biomechanically weak.[8]

The advantage of vascularised bone is the superior healing in compromised settings. Pedicle flaps from the ribs have been used for thoracolumbar spinal reconstruction.[9,10] Fibula provides straight tubular bone with excellent strength.[11,12] Vascularised fibula transfer has been used to stabilize the cervical spine after tumour resection,[13] severe kyphosis caused by degenerative arthritis,[14] neurofibromatosis,[15] chronic osteomyelitis[16] and in a case of cervical spinal osteoradionecrosis and osteomyelitis following radiotherapy.[17] In a recent paper, Winters *et al.*[18] have described the use of free vascularised fibula in 21 patients for the reconstruction of spinal defects of which four cases were of cervical spine. Krishnan *et al.*[19] have reported a case using free fibula for ventral spinal fusion at multiple levels. Other osteocutaneous donor sites such as iliac crest, scapula and radius have the disadvantages of excess soft tissue bulk and inadequate bone stock. Fibular skin paddle is thin and favours speedy resumption of swallowing.

Though there are a few earlier reports of this reconstructive technique, our case stands out as it demonstrates the complexity in the management of recurrent skull base chordomas, and the aptness of vascularised fibula osteocutaneous flap in the composite reconstruction of pharyngeal wall and cervical spine.
